# Stress-Tolerant Endophytic Isolate *Priestia aryabhattai* BPR-9 Modulates Physio-Biochemical Mechanisms in Wheat (*Triticum aestivum* L.) for Enhanced Salt Tolerance

**DOI:** 10.3390/ijerph191710883

**Published:** 2022-09-01

**Authors:** Mohammad Shahid, Mohammad Tarique Zeyad, Asad Syed, Udai B. Singh, Abdullah Mohamed, Ali H. Bahkali, Abdallah M. Elgorban, John Pichtel

**Affiliations:** 1 Plant-Microbe Interaction and Rhizosphere Biology Lab, ICAR-National Bureau of Agriculturally Important Microorganisms (NBAIM), Mau 275103, India; 2 Department of Agricultural Microbiology, Faculty of Agricultural Sciences, Aligarh Muslim University, Aligarh 202002, India; 3 ICAR-National Bureau of Agriculturally Important Microorganisms (NBAIM), Mau 275103, India; 4 Department of Botany and Microbiology, College of Science, King Saud University, P.O. Box 2455, Riyadh 11451, Saudi Arabia; 5 Research Centre, Future University in Egypt, New Cairo 11835, Egypt; 6 Natural Resources and Environmental Management, Ball State University, Muncie, IN 47306, USA

**Keywords:** endophytic microbes, abiotic and biotic stress, *Priestia aryabhattai*, wheat seedlings, growth improvement, electrolyte leakage, antioxidant enzymes

## Abstract

In efforts to improve plant productivity and enhance defense mechanisms against biotic and abiotic stresses, endophytic bacteria have been used as an alternative to chemical fertilizers and pesticides. In the current study, 25 endophytic microbes recovered from plant organs of *Triticum aestivum* L. (wheat) were assessed for biotic (phyto-fungal pathogens) and abiotic (salinity, drought, and heavy metal) stress tolerance. Among the recovered isolates, BPR-9 tolerated maximum salinity (18% NaCl), drought (15% PEG-6000), and heavy metals (µg mL^−1^): Cd (1200), Cr (1000), Cu (1000), Pb (800), and Hg (30). Based on phenotypic and biochemical characteristics, as well as 16S rDNA gene sequencing, endophytic isolate BPR-9 was recognized as *Priestia aryabhattai* (accession no. OM743254.1). This isolate was revealed as a powerful multi-stress-tolerant crop growth promoter after extensive in-vitro testing for plant growth-promoting attributes, nutrient (phosphate, P; potassium, K; and zinc, Zn) solubilization efficiency, extracellular enzyme (protease, cellulase, amylase, lipase, and pectinase) synthesis, and potential for antagonistic activity against important fungal pathogens viz. *Alternaria solani*, *Rhizoctonia solani*, *Fusarium oxysporum*, and *Ustilaginoidea virens*. At elevated salt levels, increases were noted in indole-3-acetic acid; siderophores; P, K, and Zn-solubilization; ACC deaminase; and ammonia synthesized by *Priestia* *aryabhattai*. Additionally, under in-vitro plant bioassays, wheat seedlings inoculated with *P.* *aryabhattai* experienced superior growth compared to non-inoculated seedlings in high salinity (0–15% NaCl) environment. Under NaCl stress, germination rate, plant length, vigor indices, and leaf pigments of wheat seedlings significantly increased following *P.* *aryabhattai* inoculation. Furthermore, at 2%-NaCl, *B.* *aryabhattai* greatly and significantly (*p* ≤ 0.05) decreased relative leaf water content, membrane damage, and electrolyte leakage compared with the non-inoculated control. Catalase, superoxide dismutase, and peroxidase activity increased by 29, 32, and 21%, respectively, in wheat seedlings exposed to 2% NaCl and inoculated with the bacteria. The present findings demonstrate that endophytic *P.* *aryabhattai* strains might be used in the future as a multi-stress reducer and crop growth promoter in agronomically important crops including cereals.

## 1. Introduction

Several endophytic microbes residing within plant tissue often support the host by delivering a suite of nutrients and growth factors. Endophytes are a group of plant growth-promoting microorganisms that reside within plant tissue without harming their host [1]. These microbes are endowed with multiple beneficial properties including substantial disease resistance, alleviation of soil saturation and drought stress, and increased competition and furnish these benefits to the host plant [2,3]. Endophytes support their host plants by releasing antibiotics/antimicrobials or extracellular enzymes that protect them from disease and insect/nematode infestation [4].

Endophytes have been discovered with the ability to facilitate plant growth by delivering essential nutrients; synthesizing phytohormones, iron-chelators, siderophore, and enzymes; and defending the plant from attack by pathogens.

Endophytic bacteria support the plant growth by: (i) solubilizing mineral nutrients such as phosphorous (P), potassium (K), and zinc (Zn) [5]; (ii) synthesizing plant hormones such as indole-3-acetic acid (IAA) [6]; (iii) producing ammonia (NH_3_) and hydrogen cyanide (HCN) [7], iron (Fe)-chelating compounds (e.g., siderophores) [8], and anti-microbial substances [9]; and (vi) releasing extracellular enzymes.

Furthermore, endophytes have evolved powerful mechanisms to survive within plant tissue, making them one of the most potent and reliable instruments for biotic stress relief without causing environmental disruption [10,11]. Numerous studies have demonstrated that cereal crops including wheat (*Triticum aestivum* L.) are hosts to various species of halotolerant endophytic microbes having a wide range of functional properties that promote crop growth and impart protection from multiple environmental stressors, both biotic and abiotic.

The genus *Priestia* (previously known as *Bacillus*) belongs to the Bacillaceae family of Bacillales order, and includes Gram-positive (and a few Gram-negative) rod-shaped bacteria. Several genera of endophytic isolates including *Bacillus* species associated with the root and stem of cereal host crops have been evaluated for their ability to impact plant growth and prevent stress from abiotic stimuli (e.g., salinity, heavy metals, and drought) and protect the crops from fungal attack.

Endophytic bacteria can be divided into two categories: (i) obligatory, i.e., those that spend their entire lives within plant tissue; and (ii) facultative, i.e., those that thrive in soil, on plant surfaces and inside plants [12]. In the current work, *P. aryabhattai* strain BPR-9 appears to be a facultative endophytic microbe capable of improving wheat development by solubilizing mineral nutrients (P, K, and Zn), generating indole-3-acetic acid (IAA), and releasing ACC deaminase, ammonia and, siderophores.

The isolation of bacterial strains from different organs of the wheat host demonstrates that it not only survives the growing season in the plant environment, but also operates as an endophytic PGP mechanism. A facultative endophytic lifestyle likely results in more competitive bacteria than common rhizospheric bacteria, since they may be inoculated into seeds and disseminated systemically throughout the plant. Other investigations have found that inoculating plants with facultative endophytic *Bacillus* strains imparts positive effects on plant growth and nutrient absorption [13].

Salinity is one of the more notorious variables that limit agricultural productivity, as it affects seedling germination, plant physiological development, and, ultimately, crop output. Wheat (*Triticum aestivum* L.) is an important staple crop and globally the productivity and nutritional value are both reduced by salt stress [14]. After being inoculated with PGPR, however, plant physiological, enzymatic and biochemical changes occur which act to relieve both salt and drought stress [15].

To cope with salinity stresses in edible crops, halotolerant soil bacteria are a highly promising option. By producing various growth-regulating PGP substances, halotolerant soil bacteria may possibly counteract the undesirable impacts of salinity. The mechanism of salt-tolerance and its amelioration of PGPR include: (i) release of plant-growth-regulation hormones (IAA, ABA, GA, and cytokinins); (ii) production of ACCD enzymes which reduce the level of ethylene in root tissue; (iii) release of extra-polymeric substances, such as exopolysaccharides (EPSs) and extracellular enzymes; and (iv) induced systemic resilience (ISR) to phyto-fungal infection by antifungal metabolites produced by PGPR. The antioxidant genes that play a role in maintenance of reactive oxygen species (ROS) levels in plants subjected to severe salt stress are expressed by halotolerant PGP bacteria, showing the importance of NaCl-tolerant microorganisms in free radical scavenging in saline circumstances. Furthermore, because enzymes are engaged in neutralization of free radicals, the quantum of free enzymes is reduced, resulting in decreased enzyme production, as well as having a role in plant growth promotion. Another rationale for PGPR-inoculated growth augmentation is that salt-tolerant soil microbes assist plants under NaCl stress by employing ACC deaminase to break down increased ethylene hormone levels, which limits plant growth in stressful environments.

Several studies have discovered that endophytic bacteria are important in alleviating salt stress in a range of agricultural plants [16,17]. For instance, PGP endophytic bacterium *Cronobacter* sp. strain YSD YN2 enhanced the germination, growth morphology and antioxidant activity, and reduced MDA content in plants as reported by Wang et al. (2022). Likewise, the plant growth promotion and phytopathogen inhibition potential of halotolerant endophytic isolates *Arthrobacter* and *Pseudomonas* species have been reported [18]. Lu et al. claimed that *Pantoea ananatis* D1, an endophytic bacterium, ameliorated NaCl-induced oxidative stress and enhanced growth parameters of rice seedlings by synthesizing multifarious PGP metabolites [19]. An endophytic strain, *Bacillus subtilis* BERA-71, increased NaCl tolerance in *Cicer aritienum* (L.) by regulating ROS-scavenging antioxidative enzymes (POD, CAT, SOD and GR) and non-antioxidant enzymatic activities [20].

The use of salinity-relieving bacteria in saline-induced agricultural operations is a green option for enhancing crop output, and more research into how they work and affect the plant system is needed. Taking these factors into account, we recovered salt-tolerant endophytic bacteria which were identified as *Priestia aryabhattai*. This is a Gram-positive, rod-shaped species (residing inside/outside several hosts), which can synthesize numerous essential growth-promoting metabolites. *Priestia* (formerly known as *Bacillus*) is a new species designation of the Bacillaceae family of the Bacillales order.

To date, few reports exist of stress-tolerating *Priestia* species inhabiting plant tissue and their potential role in enhancing plant growth in salinity-stressed conditions. Thus, a thorough study of agricultural endophytes can aid in elucidating their capabilities and prospects for enhancing crop development and health in a sustainable and environmentally-friendly manner. The aim of the current study was to address the objectives: (i) isolation of endophytes from tissue of wheat crops; (ii) evaluation of abiotic (salinity, drought, heavy metal) stress tolerance, antagonistic potential (against fungal pathogens), and extracellular enzymes production by endophytic isolates; (iii) assessment of essential PGP metabolite production including indole-3-acetic acid (IAA), siderophores, NH_3_, HCN and ACC deaminase, and mineral (P, K and Zn) solubilization by endophytes; (iv) identification (using 16S rRNA) of endophytic strain BPR-9 and assessment of synthesis of PGP substances under saline conditions; (v) evaluation of inoculation impact of *P. aryabhattai* strain BPR-9 on germination, length and vigor indices of *T. aestivum* (L.) seedlings; and (vi) determination of chlorophyll *a* and *b*, carotenoids, relative leaf water content (RLWC), electrolyte leakage (EL) and antioxidant enzymes in salt-treated and *P. aryabhattai* strain BPR-9-inoculated wheat seedlings.

## 2. Materials and Methods

### 2.1. Sample Collection

For the isolation of endophytic microbes, fresh and vigorous leaf and stem parts of *Triticum aestivum* L. (wheat) plants cultivated in agricultural fields were collected. Debris, soil particles, and microbes adhering to the outer surface of plant samples were removed.

### 2.2. Sterilization of Plant Samples

Plant stems were first washed in sterile distilled water (DW) before being immersed in 70% ethanol. Using a sterilized blade, stem parts were dissected under aseptic conditions, and the dissected pieces submerged in 70% ethanol for 2 min, following which they were rinsed with sterile DW several times. Stem pieces were subsequently immersed in Na hypochlorite (NaOCl) solution (4%) twice for 5 min, then rinsed twice with sterile double distilled water (DDW). The samples were then rinsed four to five times with distilled sterile (DS) water to eliminate any residue before being blot dried for further usage [21]. The sterilized plant samples were used for microbial isolation.

### 2.3. Isolation and Purification of Endophytic Bacteria

To allow endophytic bacteria to proliferate, stem portions (1–3 mm long) were placed on to the surface of nutrient agar (NA) medium and cultured at 37 °C for 1–7 days. By streaking on NA plates, bacterial colonies of various morphologies and growth patterns were isolated and stored on slants of same medium at 4 °C for later use.

### 2.4. Abiotic Stress Tolerance Assay

Cells were grown in trypticase soy broth (TSB) with varying concentrations of PEG-6000 solution (0, 2, 5, 10, and 15%) to study the ability of endophytic isolates to tolerate drought stress [22]. Furthermore, the salt tolerance properties of endophytic isolates were determined by cultivating cells in Luria Bertani (LB) broth amended with various concentrations of NaCl (0, 50, 100, 200, and 400 mM) and measuring optical density (OD). The capacity of isolates to tolerate heavy metals (HMs) was tested by adding salts of cadmium (Cd), chromium (Cr) copper (Cu), and lead (Pb) on nutrient agar (NA) at concentrations ranging from 25–1000 μg mL^–1^ [23].

### 2.5. Antifungal Activity (Dual Culture Assay)

Using a modified dual culture (cross-streak) method, the antagonistic activity of endophytic bacterial strains against virulent phytopathogens (*Alternaria solani*, *Rhizoctonia solani*, *Fusarium oxysporum* and *Ustilaginoidea virens*) was examined in vitro [24]. On potato dextrose agar (PDA) plates, a 5-mm disc of freshly cultured fungal cultures was implanted with a bacterial culture introduced on one side of the fungal plug. As controls, plates containing only fungal plugs were employed. Percent inhibition of pathogenic fungus was calculated after 7 days of incubation at 28 ± 2 °C using the formula:

Zone of inhibition = C − T

Percent inhibition (PI) = [(C − T)/C] × 100

where C is the control (without bacterial culture) and T is the treatment with bacterial culture.

### 2.6. Extracellular Hydrolytic Enzymes Production by Endophytic Isolates

Production of extracellular hydrolytic enzymes (cellulose, protease, amylase, lipase, etc.) was measured by growing bacterial endophytes on nutrient agar (NA) medium supplemented with a specific substrate. Substrates employed for amylase, cellulase, protease, and lipase were soluble starch (1%), carboxymethylcellulose; CMC (1%), casein (1%), and tributyrin, respectively. For amylase, cellulose, and protease detection, Petri plates were flooded with iodine reagent, Congo red, and Coomassie Brilliant Blue (CBB), respectively. When clear zone development showed positive enzyme activity, the difference between the diameter of the zone and the colony was measured in mm.

### 2.7. Screening of Bacterial Isolates for PGP Traits

#### 2.7.1. Synthesis of Indole-3-Acetic Acid (IAA) Estimation

Bacterial PGP characteristics were assessed using freshly (24-h) cultivated bacterial cells (10^6^ CFU mL^–1^). To assess the synthesis of indole-3-acetic acid (IAA), endophytic bacterial isolates were cultured in LB broth supplemented with L-tryptophan. The broth was autoclaved in glass test tubes that included 5.0 g NaCl, 10.0 g tryptone, 5.0 g yeast extract, and 1.0 g tryptophan. The endophytic isolates were introduced into the broth and kept at 28 ± 2 °C for 3–4 days in a shaker incubator at 120 rpm. The resultant pink color was read at 530 nm using a UV-vis-spectrophotometer [25,26].

#### 2.7.2. ACC Deaminase Production

The method of Penrose and Glick [27] was used to assess activity of ACC deaminase. Trypticase soy agar (TSA) medium containing 0.85 M NaCl was used to cultivate bacteria. Filter- sterilized (0.2 m) and frozen at −20 °C was a 0.5 M ACC solution. Freshly-grown endophytic isolates were streaked on NFb medium mixed with 3.0 mM ACC and incubated. The capacity of a strain to use ACC was tested by keeping the strain in a controlled environment with no N supply. The quantity of α-ketobutyrate generated, when the ACC deaminase cleaves ACC, was measured [27].

#### 2.7.3. Solubilization of Minerals (P, K, and Zn)

Phosphorus solubilization was measured using Pikovskaya’s (PVK) agar plates. A bacterial culture was inoculated in the center of each plate. After plates were incubated at 28 °C for 7 days, a halo zone (i.e., zone of solubilization) formed by the bacterial strain was measured [26,28]. Endophytic isolates were tested for their ability to dissolve potassium (K) by growing in Alexandrov culture media. A volume of 10 µL of bacterial culture was placed on Alexandrov agar medium and incubated for 3 days at 30 °C. Colony diameter was measured after incubation. Using a modified Pikovskaya (PVK) medium, the endophytic isolates were tested for their capacity to dissolve zinc (Zn). Then, 10 µL of overnight culture were placed onto plates containing PKV medium and cultured at 30 °C for 72 h. Colony diameter and halo zones were measured. Using the clear zone diameter/colony diameter formula, the solubility index was computed [29].

#### 2.7.4. Siderophore Production

To examine siderophore production by the endophytic isolates, 60.5 mg chrome Azul S (CAS) was dissolved in 50 mL water and introduced to a 10 mL Fe(III) solution. The solution was gently mixed with 40 mL of hexadecyl trimethyl ammonium (C_19_H_42_BrN)-containing water, and the broth was autoclaved. Freshly cultured endophytic isolates were inoculated into the center of blue agar plates and incubated for 48–72 h at 28 °C. Siderophore formation was indicated by a yellow to orange zone encircling the bacterial colonies [30,31].

#### 2.7.5. HCN and Ammonia Production

Hydrogen cyanide (HCN) generation by the isolates was assessed using LB agar plates supplemented with 4.4 g glycine L^−^^1^ after steeping in a 0.5 percent picric acid and 2% sodium carbonate (Na_2_CO_3_) solution. The insides of the upper lids of Petri plates were lined with Whatman No. 1 filter paper. Streaked plates were covered with Parafilm and incubated for five days at 28 °C [32]. Filter paper which changed from yellow to orange-brown indicated successful HCN generation. Bacterial cultures were cultivated in peptone water and incubated with 0.5 mL Nessler’s reagent. If the bacteria responded well to ammonia production, the medium changed from brown to yellow [33].

### 2.8. Identification of Endophytic Isolate BPR-9

To identify the endophytic isolate using 16S rRNA, complete genomic DNA was extracted from a pure strain using the method previously described [34]. In a thermo-cycler, forward (27 F) and reverse (1492R) primers were used to amplify the region of the 16S rDNA gene. Gel electrophoresis (0.8%) on agarose gel was used to assess the purity of DNA. On 1.2% agarose gel with a 1Kb DNA ladder (Promega) and a gel documentation device, PCR results were examined and documented. The 16S rRNA gene sequences of the endophytic strain were compared to strain sequences accessible on the NCBI database. To gain an accession number, BLAST was performed, and gene sequences were submitted to the NCBI GenBank database. Phylogenetic analysis and tree construction were carried out using the MEGA 7.0 program (Mega Limited, Paris, France).

### 2.9. PGP Attributes under Salt Stress

Under NaCl stress, essential PGP metabolites (IAA, ACC deaminase, siderophores), P solubilization, and NH_3_ and HCN produced by *P. aryabhattai* BPR-9 were assessed by growing freshly cultivated (24 h) bacterial cells (10^6^ CFU mL^–1^) in their respective media supplemented with increasing (0, 2, 5, 7, 10, 12, and 15%) salt concentrations (For detailed methodology, see Section 2.7.1, Section 2.7.2, Section 2.7.3, Section 2.7.4 and Section 2.7.5).

### 2.10. Bio-Inoculation Impact of Endophytic P. aryabhattai BPR-9 on Wheat Seedlings under Salinity Stress: Greenhouse Experiments

#### Planting, NaCl Treatment, Bacterial Inoculation, and Assessment of Germination and Growth

The endophytic strain BPR-9 was chosen for a greenhouse study based on PGP characteristics, anti-phytopathogenic potential, and abiotic stress resistance. Seeds were submerged under running tap water and then surface-sterilized with 1 percent sodium hypochlorite (NaOCl) for 5 min. The PGPR strain (inoculum density of 10^8^ CFU mL^−^^1^) was cultivated overnight and suspended in PBS for seed treatment. Surface-sterilized seeds were immersed in bacterial culture and NaCl solution (without bacteria for control) for 12 h. Every third day, salt stress was induced in experimental plastic pots by irrigating with varying levels of salt (0–15% NaCl) solution. The experiment was conducted in three replicates (*n* = 3). In the greenhouse, five seedlings/pots were planted. Biological attributes such as length and weight (fresh and dried) of shoots and roots were measured at 30 days after seeding.

### 2.11. Assessment of Salinity-Stress Related Parameters

#### 2.11.1. Photosynthetic Pigment Estimation

Accumulation of leaf photosynthetic molecules (chlorophyll a and b, and carotenoids) in NaCl-treated/untreated and bacteria-inoculated wheat foliage was assessed using Arnon’s [35] and Kirk and Allen’s [36] methods.

#### 2.11.2. Relative Leaf Water Content (RLWC) and Membrane Damage Index (MSI) Assessment

To obtain turgid weight, leaf samples were chopped, weighed, and kept in DDW for 3 h for measurement of relative leaf water content (RLWC). Samples were subsequently oven-dried for 24 h at 80 °C until they reached a constant weight [37]. The RLWC was calculated by:

RLWC (%) = Fresh weight − Dry weight × 100

Turgid weight − Dry weight

#### 2.11.3. Determination of Electrolyte Leakage (EL)

The technique of Lindén et al. [38] was used to determine electrolyte leakage (EL) in salt-treated and *P. aryabhattai*-inoculated wheat seedlings. Leaves were cut into 5 mm lengths and inserted in test tubes containing 10 mL deionized water. The electrical conductivity (EC1) of the media was determined after shaking the tubes at 30 °C for 4 h. The test tubes were then autoclaved at 121 °C for 20 min and cooled to 25 °C before measurement of electrical conductivity (EC2). The following formula was used to determine electrolyte leakage:

Electrolyte leakage = (EC1/EC2) × 100

#### 2.11.4. Antioxidant Enzymatic Activity

To determine antioxidant enzymatic activity, 0.5 g of fresh wheat seedlings were extracted in a buffer which contained 5.0 mL of 50 mM PSB and 1% polyvinylpyrrolidone (PVP) [28,39]. The resultant supernatant was utilized for the antioxidant test after centrifugation of the crude extract at 10,000× *g* for 15 min at 4 °C. The activity of catalase (CAT) was evaluated by measuring decrease in H_2_O_2_ absorbance at 240 nm. For this, 100 mL enzyme extract, 50 mM phosphate buffer (pH 7.8), 0.1 mM EDTA, and 12.5 mM H_2_O_2_ were mixed and made up to 3.0 mL. The extinction coefficient of 0.04 mM^−1^ at 240 nm was used to determine CAT activity. The activity of peroxidase (POX) in the extract was measured using a modified version of Kar and Mishra’s technique [40].

### 2.12. Statistics Analysis

All tests were conducted in triplicate (*n* = 3) in a completely randomized block design (RBD). One-way ANOVA was used to establish statistical significance between each treatment effect. Duncan’s multiple range test (DMRT) was used to compare mean values post hoc at a 5% probability level.

## 3. Results and Discussion

### 3.1. Recovery of Endophytes and Their Cultural Characterization

In the present investigation, we attempted to discern the essential mechanisms employed by halotolerant endophytic PGPR strains to reduce salinity stress in wheat plants via modulation of plant defense systems. A total of 25 endophytic isolates were recovered from different parts of wheat. The isolates were further characterized based on biochemical assays and utilization pattern of different carbon sources (and demonstrated variable responses). The screened halotolerant organisms were identified using 16S rRNA sequences and a comparison of the 1500-bp 16S rRNA gene sequence was submitted to BLAST to validate relatedness to other bacterial strains. The genomes of bacteria labelled as *Priestia aryabhattai* (accession No. OM743254.1) in the GenBank database were closely linked (99.9 percent nucleotide identity) to the isolate BPR-9 (1485 base pair).

### 3.2. Abiotic Stress Tolerance among Recovered Endophytic Isolates

Endophytic bacteria are inherently adapted to survival in a variety of environments, including drought salt and heavy metal (HM) stress, and assist their hosts in coping with these pressures. Salinity is a significant problem in many modern agricultural systems; salinity can adversely affect the growth, biological characteristics, and yield of a wide range of economically important crops [41]. To address these difficulties, we looked for a salt-tolerant endophytic PGPR strain that could be utilized as an inoculant for increasing the development and productivity of salt-tolerant crops. A total of 25 endophytic isolates were recovered and their capacity to tolerate salinity was tested. The diversity and number of endophytic bacteria found in different tissues of wheat plants varied markedly. The isolates demonstrated, overall, significant resilience to abiotic stresses. The majority of screened endophytes obtained from wheat hosts displayed drought (15% PEG) and salt (12% NaCl) resistance. The pattern of NaCl tolerance among isolates varied from 5% NaCl (BPR-23) to 18% (BPR-9). Increased PEG concentrations demonstrated negative impacts on bacterial development. Among the recovered isolates, BPR-9 showed maximum tolerance to drought stress (18% PEG) (Table 1). Isolates BPR-19 and BPR-25 were unable to tolerate the PEG concentrations tested. Recently, abiotic stress-tolerant endophytic strains were recovered from wheat seedlings. Additionally, Al-Shwaiman et al. reported that multi-stress tolerant *Beijerinckia fluminensis* BFC-33 tolerated variable concentrations of heavy metals [42].

Soil pollution by heavy metals (HMs) is one of the world’s most serious agricultural and environmental issues [43]. Accumulation of HMs including cadmium (Cd), copper (Cu), chromium (Cr), mercury (Hg), and others in agricultural soils can pose serious health risks to humans, soil, and ecosystems. Table 1 summarizes the heavy metal (Cd, Cr, Cu, Pb, and Hg) tolerance of the bacterial isolates at various concentrations (0–1200 µg mL^−1^). Several studies have found that endophytic bacteria isolated from different plant organs can aid plant development and increase uptake of HMs by regulating various mechanisms [44,45,46]. Additionally, the majority of endophytic bacteria produce indole-3-acetic acid (IAA) and siderophores and solubilize minerals, all of which point to their potential benefits to plant growth promotion and tolerance to HMs. As a result, the goal of this study was to examine the HM tolerance of endophytic strains linked with the wheat host. The current findings support previous research demonstrating the important role of endophytic microbes in plant growth promotion and remediation of HM pollution [10,47]. In accordance with the current study, Long et al. [48] isolated two metal-tolerant endophytic bacterial isolates (*Bacillus* sp. C9-3 and *Microbacterium* sp. D2-2).

### 3.3. Antifungal Activity of Recovered Isolates

**Figure 1:**
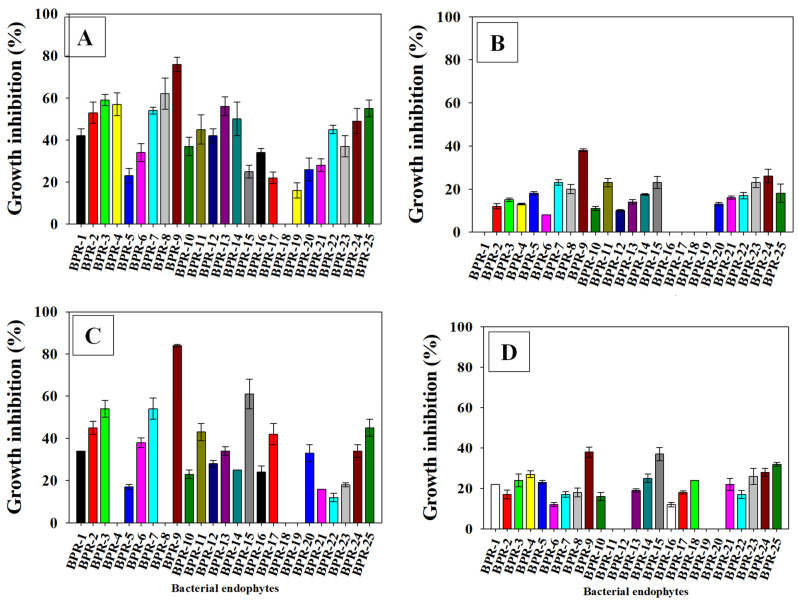
Antifungal activity of recovered endophytic bacterial isolates against major fungal phytopathogens: (**A**) *Alternaria solani*; (**B**) *Ustilaginoidea virens*; (**C**) *Fusarium oxysporum*; and (**D**) *Rhizoctonia solani*. Each bar or point represents the mean of five replicates (*n* = 5). Corresponding error bars represents standard deviation (S.D.) of the five replicates.

Antifungal activity of endophytes is diverse, and may be associated with the release of secondary compounds (metabolites). In the current work, recovered endophytes were assessed for their antagonistic potential against the major phyto-fungal pathogens viz., *Alternaria solani*, *Fusarium oxysporum*, *Rhizoctonia solani*, and *Ustilaginoidea virens*. These phytopathogens were chosen as test organisms for antifungal fungal assays because of their varied host range, rapid growth, and potential to inflict significant economic damage to different groups of agricultural crops. Cross-streak (dual culture) methods revealed that the antagonistic behavior of endophytic isolates varied (Figure 1). For example, BPR-9 suppressed the growth of phytopathogens in the following order: *Fusarium oxysporum* (85%) > *Alternaria alternata* (76%) > *Rhizoctonia solani* (38%) > *Ustilaginoidea virens* (32%) (Figure 1A–D). The development of structurally distinct antimicrobial compounds and antibiotics having wide-spectrum antagonistic properties could be one of the most significant properties of these strains contributing to biocontrol [49]. This study did not investigate the exact mechanism of action of the endophytic strains; however, production of siderophores and hydrolytic enzymes (protease, amylase, lipase, and cellulose) reflects bacterial competitiveness, which may be beneficially paired with plant protection and PGP features to boost plant development. Indigenous microorganisms associated with plants in agro-desert habitats have been shown to possess substantial phytopathogen antagonistic capability [50]. Accordingly, strain *B. amyloliquefaciens* can withstand diverse abiotic stressors and exhibits biocontrol activity, among other PGP characteristics [51].

### 3.4. Production of Extracellular Enzymes

**Figure 2:**
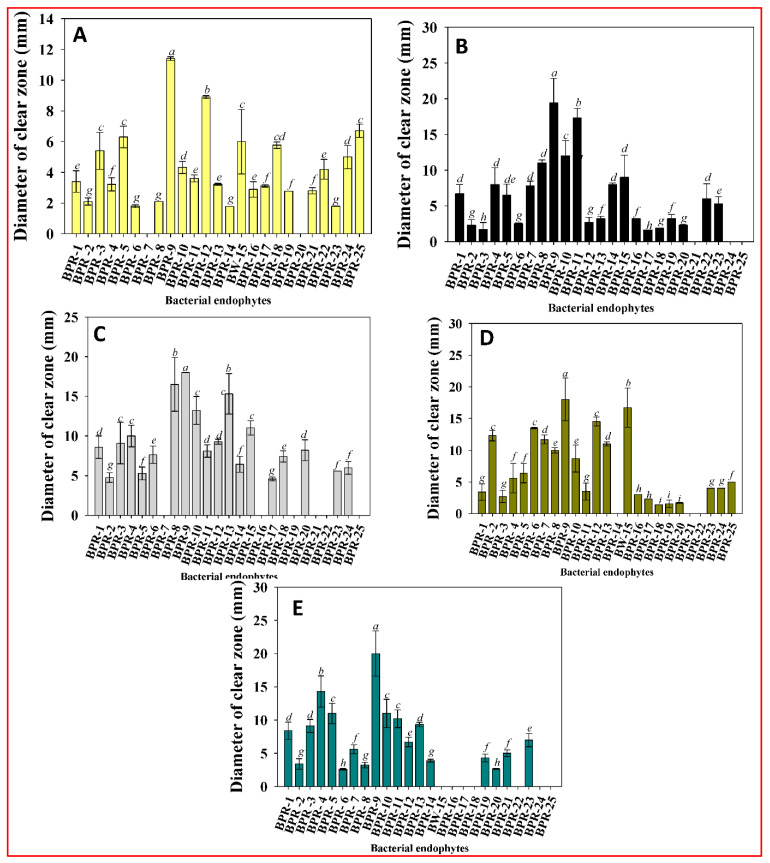
Clear zones produced as a result of extracellular enzyme activity from bacterial endophytes: (**A**) protease; (**B**) cellulase; (**C**) amylase; (**D**) lipase; and (**E**) pectinase. Each bar represents the mean of five replicates (*n* = 5). Corresponding error bars represents standard deviation (S.D.) of five replicates. Letters a, b, c, d etc. in figure depicts that mean values are significantly different from each other according to Duncan’s multiple range test (DMRT).

Endophytic microbes have been shown to support plant growth by a variety of mechanisms. Endophytes enter and remain in the host via several methods; one involves production of hydrolytic enzymes, which help bacteria penetrate root hairs and lateral roots by hydrolyzing cell wall components. From a biological control perspective, the presence of hydrolytic enzymes linked to PGPRs is a requirement for identifying the most successful strains. Their presence suppresses the development of fungal phytopathogens and strengthens plant tolerance to disease [52]. Many soil bacteria, including endophytes, use such lytic enzymes as major components of their defense and attack mechanisms. These enzymes are used to combat phytopathogen-induced crop disease. Chitin, an insoluble polymer found in the cell walls of higher fungi, gut walls of insects, and worms, can be hydrolyzed by microbial chitinases, ultimately breaking down their cell walls. Furthermore, chitin and glucan oligomers generated during the breakdown of the fungal cell wall function as elicitors, triggering a variety of plant defensive systems. Considering their significance in biological control of fungal pathogens, the endophytic isolates were assessed for production of extracellular enzymes. Synthesis of protease (92%), cellulase (84%), amylase (76%), lipase (88%), and pectinase (72%) varied among the total (*n* = 25) endophytic isolates. Among the endophytes (*n* = 25), strain BPR-9 produced the maximum amount of protease (18.0 mm), cellulase (11.4 mm), amylase (20 mm), lipase (16.5 mm), and pectinase (19.4 mm) (Figure 2A–E). Various bacteria, including *Pseudomonas stutzeri*, generate these enzymes, which can lyse the fungus *Fusarium* sp. [53]. Bhutani et al. recovered forty-seven (47) endophytic isolates from *Cajanus cajan* and *Vigna radiata* plants and among them, nine isolates potentially synthesized extracellular enzymes [54].

### 3.5. Essential Plant Growth Promoting (PGP) Metabolites of Recovered Endophytes

It has been observed that halotolerant PGPR promote plant development while also reducing salinity stress. Plant development in saline environments is linked to the capacity of halotolerant PGPR to produce phytohormones. Plant stress defense responses are controlled by these phytohormones, which impact cell wall elongation (IAA), cell division (CK), germination (gibberellin), and stress tolerance (gibberellin). According to previous research, phytohormones released in response to salt stress aid in plant survival and tolerance to various abiotic challenges [28,55]. Considering the significance of bacterially- synthesized plant growth regulating essential metabolites, we assessed the PGP activities of recovered endophytic isolates. The majority of the endophytes were able to produce phytohormones (IAA), siderophores, ACC deaminase, ammonia and hydrogen cyanide (Table 2).

Low nutrient availability limits crop development. Reduced nutrient acquisition in agricultural soil is caused by low solubility of nutrient elements such as phosphorous (P), potassium (K), and zinc (Zn) [56]. The majority of endophytic microbes recovered in this investigation converted insoluble P, Zn, and K to soluble forms as evidenced by development of a translucent halo (i.e., zone of solubilization) (Table 3). These findings support prior studies that showed that bacterial strains differ in their ability to solubilize nutrients. The development of a transparent halo zone implies the solubilization capacity of the endophytic strains, which may be attributed to a pH shift and subsequent solubilization of P, Zn, and K forms surrounding the bacteria. The current investigation reveals a wide range of endophytic microbes capable of solubilizing mineral nutrients containing phosphate, potassium, and zinc. NaCl-tolerant endophytic PGPR strain *Serratia rubidaea* solubilized phosphate and zinc forms, thus increasing the salinity tolerance of plants by improving seed germination [57]. Similarly, endophytic strains such as *Pseudomonas* sp., *Bacillus pumilus*, and *Enterobacter colacae* recovered from sugarcane showed the anti-fungal potential for solubilizing the insoluble forms of P, K, and Zn minerals [58].

### 3.6. Molecular Identification

**Figure 3:**
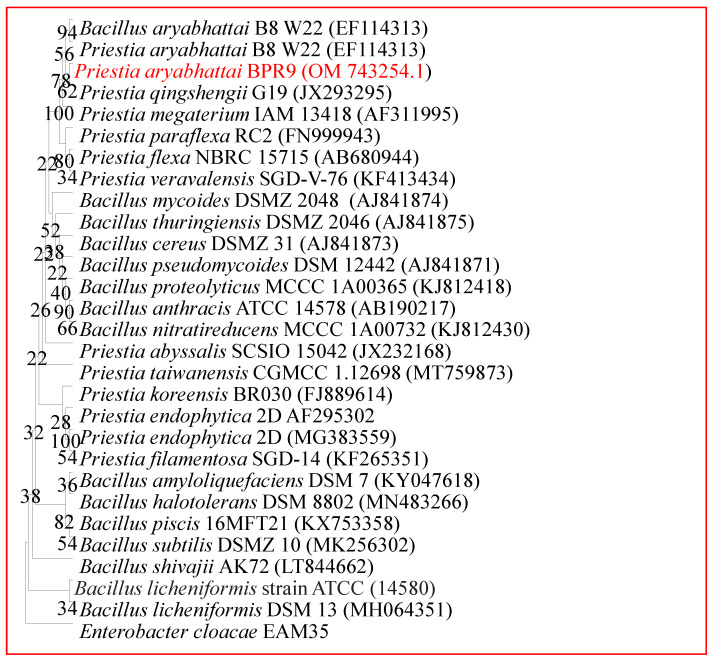
Neighbor-joined phylogenetic tree of *Priestia aryabhattai* BPR-9. The tree was constructed based on 16S rRNA partial gene sequence of selected bacteria (marked in red) and closely related phylogenetic species derived using the NCBI BLAST search tool. Sequences were aligned using the Clustal W sequence alignment tool with MEGA 7.0 software.

The endophytic bacterial isolates were also molecularly characterized for species-level identification using 16S rRNA gene sequencing. The BLAST analysis of the 16S rRNA of strains BPR-9 with the non-redundant NCBI (http://blast.ncbi.nlm.nih.gov/Blast.cgi, accessed on 20 May 2022) database revealed a 99 percent resemblance to members of the genera *Priestia*. The 16S rRNA nucleotide sequences of BPR-9 (about 1418 bp in length) were submitted to GenBank (accession No. OM743254.1). Using BLASTn, a similarity search revealed that strain BPR-9 was closely (>99.9%) related to *Bacillus aryabhattai* strain Os Enb-PLM-L62 (accession No. MN889284.1). Thus, strain BPR-9 was identified as *Priestia aryabhattai* based on its strong relatedness with *Bacillus*/*Priestia aryabhattai*. MEGA 6.0 software was used to create a phylogenetic tree (Figure 3) based on the partial sequencing of the 16S rRNA gene. Evolutionary history was inferred using the Maximum Likelihood method and Tamura–Nei model. The tree with the highest log likelihood (−7444.07) is shown. The percentage of trees in which the associated taxa clustered together is shown next to the branches. Initial tree(s) for the heuristic search were obtained automatically by applying Neighbor-Join and BioNJ algorithms to a matrix of pairwise distances estimated using the Tamura–Nei model, and then selecting the topology with superior log likelihood value. The proportion of sites where at least 1 unambiguous base is present in at least 1 sequence for each descendent clade is shown next to each internal node in the tree. In recent studies, researchers have characterized different species of *Priestia*, including *P. megaterium* [59], *Priestia* sp., and *P. endophytica* [60].

### 3.7. PGP Attributes of P. aryabhattai under Salinity Stress

#### 3.7.1. IAA, Siderophores, and ACC Deaminase

**Figure 4:**
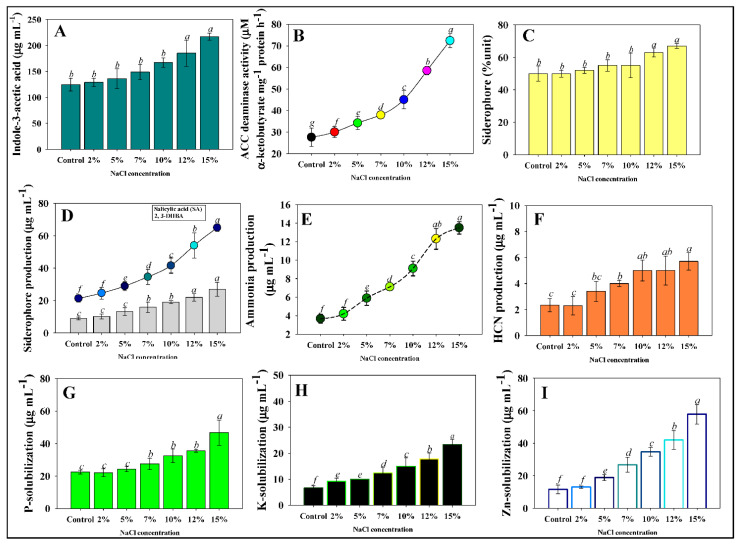
Plant growth promoting attributes of strain BPR-9 under varying levels of NaCl stress: (**A**) indole-3-acetic acid production; (**B**) 1-amino cyclopropane 1-carboxylate (ACC) deaminase; (**C**) % unit production of siderophore; (**D**) quantification of salicylic acid and 2, 3-DHBA; (**E**) ammonia and (**F**) HCN production; (**G**) phosphate, (**H**) Zn, and (**I**) K solubilization. Bar and line diagrams represent the mean of five replicates (*n* = 5). Corresponding error bars represents standard deviation (S.D.) of five replicates. Letters a, b, c, etc. represent mean values that are significantly different from each other (DMRT test).

When the PGPR strains were cultivated in untreated and salt-stressed conditions, production of growth-regulating substances was inconsistent. With an increasing dose of NaCl, growth-controlling activities increased. At 0% NaCl (control), strain BPR-9 produced 124 µg IAA mL^−1^, which increased with elevated salt level. For example, a maximum 217 μg IAA mL^−1^ (42% increase over control) was recorded with 15% NaCl (Figure 4A). The salt tolerance capability of strain BPR-9 is responsible for the increase in IAA in the presence of increased salinity. About 80 percent of soil beneficial populations release IAA, a physiologically active auxin [61]. Even under harsh settings, the IAA generated by soil bacteria regulates plant physiological and metabolic process [62]. IAA also tightens root cell walls, resulting in reduced root exudates, which stimulates PGPR development by supplying more nutrients. Several IAA-synthesizing salt-tolerant PGPR strains obtained from salinity-stressed environments have been shown to improve the growth of edible crops. For example, auxin-producing halotolerant PGPR increased salinity tolerance in wheat and improved growth performance [63]. Hidri et al. found that increasing the halotolerant PGPR strains is more likely to produce/release phytohormones accessible to plants even in salty soils [64]. The ability of halotolerant endophytic microorganisms to secrete plant hormones like IAA even under extreme salt stress is an intriguing and encouraging characteristic.

ACC deaminase synthesized by plant-beneficial soil microorganisms is another outstanding biological attribute that can significantly reduce plant ethylene levels and thus enhance plant growth in harsh environments [65]. Even when cultivated in media supplemented with various levels of NaCl, BPR-9 showed a favorable reaction to ACC deaminase. With increasing NaCl concentration, the quantity of α-ketobutyrate increased steadily (Figure 4B). From rhizosphere soil samples, several salt-tolerant bacterial strains that use ACC as their only source of nitrogen have been identified. Plants produce ethylene as a stress response, and it is closely linked to numerous environmental stressors like salinity, water deficit (drought), metal (HM) toxicity, and deficiencies of mineral nutrients [66]. Researchers have observed that several salt-resistant PGPR strains produce the same level of ACC deaminase when exposed to a harsh saline environment. The endophytic bacterium *Serratia grimesii* has been demonstrated to enhance early nodulation and growth of common bean (*Phaseolus vulgaris*) by expressing ACC deaminase exogenously [67].

Under iron (Fe)-deficient conditions, siderophores, a low molecular weight (LMW) Fe-chelating complex generated by several soil microorganisms, supplies Fe to plants [68]. Insoluble iron occurs in two forms: (i) hydroxides and (ii) oxyhydroxides, neither of which are accessible to rhizospheric microbes [69]. Release of siderophores by a PGPR strain during Fe deficiency may be beneficial as they can be employed in managing phyto-fungal-induced plant disease. With increased NaCl concentrations, bacterial strains produced more siderophores (Figure 4C,D), similar to findings for phytohormone synthesis. For example, under controlled conditions, strain BPR-9 experienced a 53.2 percent siderophore unit, which rose by 3.4 percent when exposed to 15% NaCl (Figure 4C). Panwar et al. found increased siderophore synthesis at even greater NaCl concentrations, which is similar to our findings [70]. The increasing salt concentrations showed a positive effect on ammonia (Figure 4E) and HCN (Figure 4F) produced by strain BPR-9.

#### 3.7.2. Mineral Solubilization, and HCN and NH_3_ Production

A similar tendency was seen for the P (Figure 4G), K (Figure 4H), and Zn-solubilizing (Figure 4I) activity of strain, as with other assessed growth-controlling substances. Phosphorus is the second-most essential plant nutrient after nitrogen, and is involved in practically all plant metabolic functions [71]. Plant development is significantly hampered by P deficiency [72]. Plants have access to less than 5% of total soil P; as a result, phosphatic fertilizers are administered to address P deficit and allow plants to develop optimally. In recent years, however, due to the high expense of mineral P forms and environmental issues associated with its usage, attention has shifted to natural renewable alternatives for plant P supply. Phosphorus-solubilizing microbes belonging to various genera have been considered as alternatives to costly synthetic P fertilizers in this context [73]. Due to variability in their capacity to produce certain low-molecular-weight organic acids, phosphate solubilization efficiency of beneficial soil microorganisms has varied [74].

The quantities of NH_3_ and HCN emitted by the PGPR strains were unaffected by NaCl concentration. Ammonia is produced by a variety of soil bacterial communities via amino acid decarboxylation, ammonification of nitrite, hydrolytic degradation of urea, and amino acid breakdown [75,76]. Ammonia transporters are found in some PGPR strains and are assumed to be engaged in reabsorbing NH_4_^+^ released by NH_3_ diffusion across microbial membranes [77]. Salt-tolerant endophytic PGPR strains cultivated on liquid media supplemented with salt have recently been found to produce HCN and ammonia [78,79].

### 3.8. Assessment of Stress Alleviation and Growth Promoting Potential of P. aryabhattai: In-Vitro Studies

#### 3.8.1. Germination and Biometric Parameters

**Figure 5:**
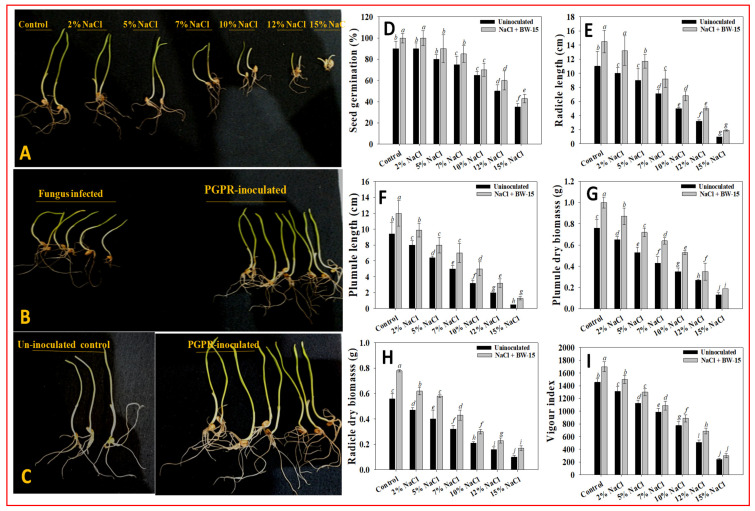
Germination of wheat seedlings in-vitro: (**A**) effect of NaCl concentration on seedling length; (**B**) fungal-infected and inoculated with BPR-9; and (**C**) non-inoculated and PGPR-inoculated seedlings. Effect of endophytic isolate BPR-9 on growth attributes of wheat seedlings under salt stress: (**D**) seed germination; (**E**) radicle length; (**F**) plumule length; (**G**) plumule biomass; (**H**) radicle biomass; and (**I**) and vigor index. Each bar or point represents the mean of five replicates (*n* = 5). Corresponding error bars represent standard deviation (S.D.) of five replicates. Different letters represent mean values that are significantly different according to DMRT test.

The ability of seeds to germinate in salt-enriched conditions serves as a practical criterion for choosing salt-tolerant endophytic microorganisms. Seed germination, seedling vigor (SVI), and seed biomass improved when treated with *P. aryabhattai* BPR-9 (Figure 5A–C). Nearly all seeds sprouted in the control; however, increased salt concentration (15% NaCl) significantly decreased both the ability of seeds to germinate and vigor indices. Application of *P. aryabhattai* BPR-9 to NaCl-treated wheat seedlings increased both germination effectiveness and vigor index (Figure 5D). In the presence of 2% NaCl, strain BPR-9 significantly enhanced percentage germination (10% increase) and vigor index (14% increase) of wheat seedlings (Figure 5). The higher germination rates and enhanced physio-biochemical traits of wheat are due primarily to seed priming with halotolerant endophytic bacteria. Improved seedling establishment is a result of higher tolerance by the seeds, which equips them to handle other environmental obstacles as well. Growth enhancement may be caused by colonization of halotolerant endophytic bacterial populations that form biofilms and secrete indole-3-acetic acid and extra-polymeric substances (i.e., exopolysaccharides and extracellular proteins) around seedling roots that are experiencing salt stress. Furthermore, applied endophytic bacteria trigger increased production of plant hormones, such as IAA, which directly activates enzyme activity. This response will increase starch absorption, encouraging early germination even in adverse settings.

#### 3.8.2. Plant Length and Dry Biomass

Endophyte-inoculated wheat seedlings grown under NaCl stress had varying lengths (Figure 5E,F) and dry biomass (Figure 5G,H). The enhanced bacterial release of plant hormones and other essential active biomolecules, which benefit plants by encouraging and interacting them with standing crops and thus increasing the root morphogenesis under unfavorable conditions, is likely the cause of improved plant biometrics [80]. Following inoculation with halotolerant endophytic strains, wheat seedlings produced longer roots, which allow them to extract more soil water under adverse conditions [81]. The overall performance of seedlings was likely increased as a result of growth-regulating substances synthesized by endophytic strains. Indole-3-acetic acid, for example, enhanced root formation, while siderophores, ACC deaminase, and NH_3_ benefited other plant developmental processes. Similarly, when cultivated under abiotic stress, several NaCl-tolerant PGPR strains have increased the growth and dry biomass of food crops, including grains. As a result, the essential growth-regulating metabolites generated by the endophytic bacteria used in the previous study may have also improved plant performance under salt stress and increased biomass accumulation. In this regard, the endophytic bacterium *Curtobacterium* sp. SAK1 increased tolerance of salinity in soybean plants [82]. Bacterial endophytes supported growth of *Arabidopsis thaliana* under salt stress [83]. Under salt-stressed conditions, endophytic bacterial strains viz., *Bacillus cereus*, and *Pseudomonas* sp. improved growth and yield attributes of soybean [84]. Ribeiro et al. revealed that endophytic *Bacillus* inoculation had a beneficial influence on plant development, biomass production, and nutrient accumulation (N, P, K) in stems [85]. Taken together, utilizing salt-stress-resistant PGPR strains to guard against abiotic pressures can contribute to food security. Application of endophytic strain *P. aryabhattai* resulted in increased biomass accumulation, indicating that salinity stress was alleviated in plants, allowing them to develop more efficiently. Halotolerant endophytic strains viz., *Curtobacterium luteum* SAK2, *Curtobacterium oceanosedimentum* SAK1, *Bacillus cereus* SA1, *Enterobacter tabaci* SA3, *Enterobacter ludwigii* SAK5, and *Micrococcus yunnanensis* SA2 resulted in a significant enhancement in growth and dry matter production of rice by increasing salt tolerance [86].

#### 3.8.3. Leaf Chlorophyll and Relative Water Content

Plant photosynthetic efficiency is reduced by salinity, which leads to generation of ROS and ultimately damages nucleic acids, extracellular proteins, and membranes. Treatment with holtolerant endophytic bacterium had a significant effect on preventing salinity-induced damage to the photosynthetic apparatus. El-Esawi et al. showed similar results, where PGPR inoculation increased photosynthetic efficiency under salinity stress [87].

Salinity inhibited growth and altered vegetative features of wheat; likewise, relative water content decreased. Salinity induced leaf curling and wilting, as well as early leaf senescence with resultant decrease in overall growth. Salinity hinders cell differentiation and the cell cycle as a consequence of osmotic and ionic stress, oxidative damage, and reduced water intake. Therefore, seed germination, tissue expansion, and various physiological processes are adversely affected, ultimately resulting in growth suppression.

**Figure 6:**
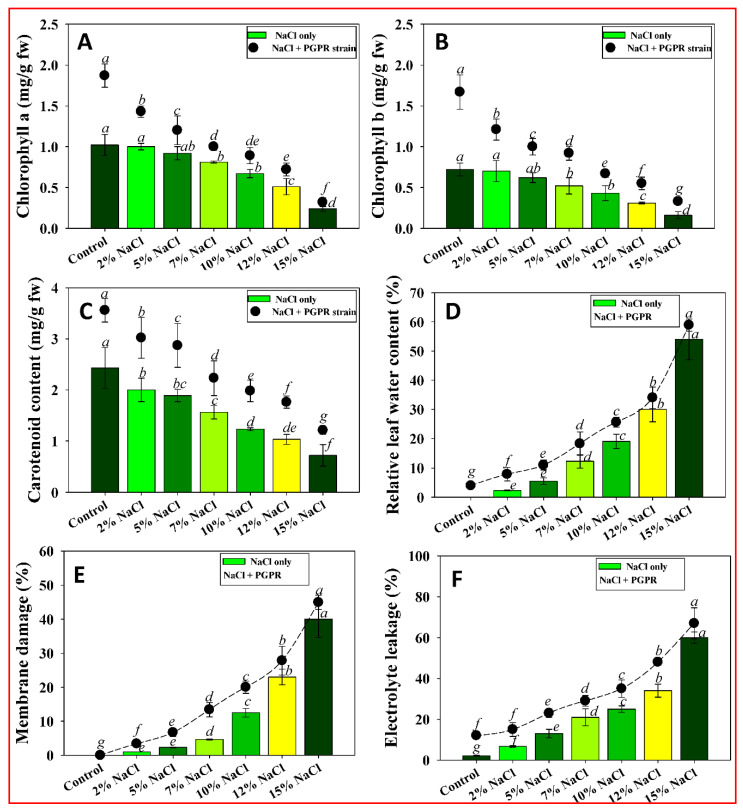
Effect of inoculation of strain BPR-9 on photosynthetic pigments of salt-treated wheat seedlings: (**A**) chlorophyll a; (**B**) chlorophyll b; (**C**) carotenoids; (**D**) relative leaf water content; (**E**) membrane damage; and (**F**) electrolyte leakage. Each bar or point represents the mean of five replicates (*n* = 5). Corresponding error bars represent standard deviation (S.D.) of five replicates. Different lower-case letters represent mean values that are significantly different according to DMRT test.

Leaf chlorophyll content is an important physiological metric for detecting plant stress. Salt stress reduces crop yield and development by inhibiting the formation of 5-aminolevulinic aminolevulinic acid, a chlorophyll precursor. Salt stress also alters chloroplast structure and down-regulates photosynthetic machinery, including chlorophyll synthesis. Plant photosynthetic processes are also regulated by carotenoid pigments, which facilitate light gathering and photo-protection [88]. According to several studies salinity lowers the chlorophyll and carotenoid concentration in a variety of plants. Application of halotolerant *P. aryabhattai* BPR-9, on the other hand, improved leaf chlorophyll content by reducing salinity stress. Strain BPR-9 resulted in increased chl a (Figure 6A), chl b (Figure 6B), and carotenoids (Figure 6C) of wheat seedlings by 33, 56, and 35%, respectively, over non-inoculated plants treated with 2% NaCl. The increased leaf pigments may be due to the action of the halotolerant endophytic isolate that enhances polyamine and antioxidant production in salt-affected plants. Similarly, endophytic PGPRs improved the growth, chlorophyll pigments, and nutrient status of salinity-induced rice seedlings [89].

Increasing salt level adversely affected leaf relative water content (RWC) of wheat seedlings. However, inoculation of endophytic isolates resulted in a considerable increase in RWC. The RWC of seedlings was maximally increased by 70.3% in BPR-9 inoculated and NaCl (2%) treated plants (Figure 6D). The use of the halotolerant PGPR strain, which presumably reduced sodium absorption in NaCl-treated *R. sativus*, could explain the decrease in RWC. The halotolerant PGPR strain *Kosakonia sacchari* relieved salinity induced-phytotoxicity in *Vigna radiata* (L.) and improved the RWC of the plants [90].

#### 3.8.4. Membrane Stability Index and Electrolyte Leakage

Plant development is primarily influenced by photosynthetic rate as well as the accessibility of H_2_O and essential minerals in the soil system. Plant strategies for decreasing salt-induced ionic imbalance and oxidative stress include inflow of K^+^ ions and outflow of Na^+^ ions. Inoculating halo-tolerant bacterial strains into salt-affected wheat seedlings reduced Na^+^ uptake while boosting K^+^ uptake, favoring the K^+^/Na^+^ ratio. Our findings match those in which *Stenotrophomonas maltophilia* inoculation with wheat boosted K^+^ absorption by 20% to 28% and reduced Na^+^ accumulation by 25% to 32% [91].

Salt stress can result in increased discharge of electrolytes as a consequence of displacement of membrane-associated Ca. Membrane permeability is, therefore, compromised, resulting in greater outflow of electrolytes from plant tissue [92]. At 15% salt stress, electrolyte leakage (EL) of wheat plants increased more rapidly than in the control group (Figure 6F). These findings contradict those of Bojórquez-Quintal et al., who discovered that salt stress increased EL and production of ROS, both of which are detrimental to plant development. Inoculation with halotolerant PGPR reduced the harmful effects of saline stress and the possibility of ion leakage of electrolytes in stress-treated plants. This is in line with findings of prior investigations [93]. Inoculation of endophytic strain BPR-9 dramatically reduced EL in NaCl-exposed seedlings. EL in wheat seedlings inoculated with PGPR strains and subjected to 2% NaCl was reduced by 40% compared to non-inoculated controls, suggesting less membrane damage.

#### 3.8.5. Antioxidant Enzymes

**Figure 7:**
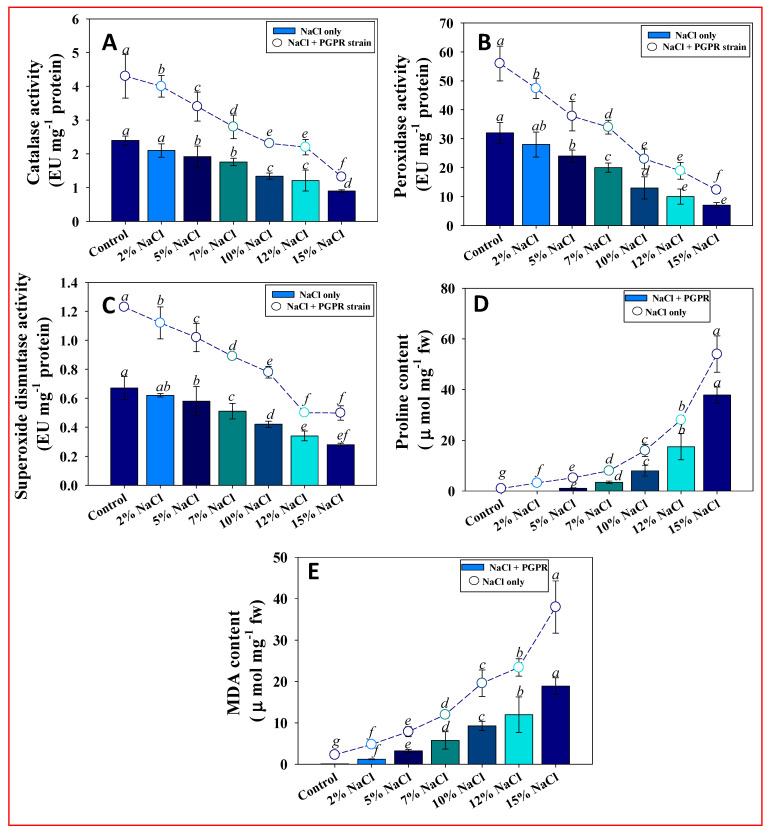
Antioxidant enzymatic activities (**A**) CAT, (**B**) POD, and (**C**) SOD; and stress biomarkers (**D**) proline, and (**E**) MDA in BPR-9 inoculated and NaCl-treated wheat seedlings. Each bar or point represents the mean of five replicates (*n* = 5). Corresponding error bars represents standard deviation (S.D.) of five replicates. Different lower-case letters represent mean values that are significantly different according to DMRT test.

Antioxidant enzymes are used by plants to minimize stress-induced oxidative damage, which is a key strategy for resistance to environmental threats. These enzymes are essential for detoxifying the harmful effects of reactive oxygen species (ROS) produced when plants are exposed to excess salt. Catalase (CAT), peroxidase (POD), and superoxide dismutase (SOD) activities increased in saline environments. (The anti-oxidative response of wheat seedlings decreased with increasing levels of salt however, inoculation of endophytic *P. aryabhattai* BPR-9 strain displayed stronger antioxidant enzymatic activity under salinity pressures than did non-inoculated plants. In comparison to non-inoculated controls, inoculation of endophytic strain BPR-9 raised CAT (Figure 7A), POD (Figure 7B), and SOD (Figure 7C) activities in wheat seedlings by 29, 21, and 32%, respectively, when subjected to 2% NaCl (Figure 7A–C). Increased enzyme activity is likely caused by bacterial inoculation. NaCl-tolerant PGPR strains (*Pseudomonas fluorescence*, *Bacillus pumilus*, and *Exiguobacterium aurantiacum*) improved wheat salinity tolerance by modulating antioxidant defense systems [94].

The halotolerant PGPR strain resulted in increased activity of antioxidant enzymes in wheat plants grown under NaCl stress, suggesting that these bacteria aid the plant in combating the harmful effects of ROS produced under salinity stress. These findings are consistent with prior research, which found that increased antioxidant enzyme activity in response to salinity stress is linked to salt tolerance. Kumari et al. likewise found that application of PGPR strains reduces oxidative damage caused by abiotic stressors such as salinity, by increasing antioxidant enzyme activity [95].

#### 3.8.6. Proline and Lipid Peroxidation under Salinity Stress

Proline buildup is a common feature of salinity-induced stress tolerance mechanisms in plants [96]. Wheat seedlings cultivated in saline conditions experienced significant foliar proline concentrations (Figure 7D). Enzymatic denaturation caused by NaCl and other stressors is reduced by proline [97]. Proline accumulation reduces the saline stress-induced decrease in antioxidant enzyme activity. Proline is considered be a hydroxyl radical scavenger as well as an osmoprotectant [98]. Proline maintains cellular activities by stabilizing proteins, membranes, and sub-cellular structures under stressful situations by removing ROS [99]. Proline buildup in plants is reported to decrease after PGPR inoculation; however, results are conflicting on the effect of PGPR on proline levels in many stressed plants. Studies have found that inoculated plants had higher proline levels than did non-inoculated plants, whereas others found that PGPR-inoculated plants had lower proline content. Different bacterial species involved, the mechanism of bacterial communication with the plant, bacterial interaction, and severity of salt stress might all contribute to these discrepancies. Proline is considered to be a stress signal in addition to being an osmoprotectant. As a result of reduced stress, inoculated plants may accumulate less proline.

Lipid peroxidation, as assessed by malondialdehyde (MDA) level, is an oxidative lipid breakdown process that produces ROS. Biological membranes are the most common candidates for lipid peroxidation; however, the process ultimately causes cell metabolism to be disrupted [100]. Increased lipid peroxidation is frequently used to evaluate plant tolerance to abiotic stresses like salinity and drought. In this work, an increase in lipid peroxidation was noted in NaCl-treated wheat foliage; maximal MDA (38 μ mol mg^−1^ fw) was measured in the presence of 15% NaCl (compared to 2.3 μmol mg^−1^ fw in the control). PGPR treatment significantly (*p* ≤ 0.05) decreased MDA levels in plants cultivated in a saline environment. For example, strain BPR-9 maximally and significantly reduced MDA level in wheat seedlings treated with 15% NaCl by 52.6% over non-inoculated controls treated with the identical salt dosage (Figure 7E). Similar effects have been found in wheat plants treated with PGPR during salinity stress (Haroon et al., 2021), as well as in PGPR-inoculated maize plants grown in saline environments. Under non-stressed and stressed circumstances, soybean (*Glycine max*) plants inoculated with halotolerant PGPR strains *Azospirillum brasilense* and *A. chrococcum* experienced substantial decrease in MDA content when compared to non-inoculated controls [101]. PGPRs decreased lipid peroxidation by limiting cellular damage.

## 4. Conclusions

The current study identified and characterized the halo-tolerant endophytic bacterium *Priestia aryabhattai* BPR-9, which was endowed with the potential for synthesizing growth-regulating attributes, tolerating numerous abiotic stress factors as well as demonstrating a wide range of anti-phytopathogenic activity. Results from this study imply that application of endophytic bacteria in agricultural fields can improve overall plant health and crop productivity, as well as increase the quality and fertility of agricultural soils. In order to support the current conclusions, further field experimental evidence is needed. By activating critical defensive mechanisms such as synthesis of osmoregulatory metabolites and activation of ROS scavenging enzymes, halotolerant endophytic bacteria benefit plants exposed to salinity. Naturally-occurring microbial flora suited to saline environments can be exploited to formulate consortia for crop inoculation, eventually leading to production of a salt-tolerant bio-fertilizer. More research, however, is required to determine how they work in real-world (i.e., field) situations. Exploring the possibility of developing more endophytes with wider host ranges could help accelerate the development of commercial inoculants.

## Figures and Tables

**Table 1:** Abiotic stress tolerance among recovered endophytic isolates.

Endophytic Isolates	Maximum Tolerance Limit (MTL)						
	NaCl (%)	PEG (%)	Heavy Metal (µg mL^−1^)				
			Cadmium (Cd)	Chromium (Cr)	Copper (Cu)	Lead (Pb)	Mercury (Hg)
BPR-1	12	10	100	50	100	200	–
BPR-2	10	12	200	100	200	400	–
BPR-3	10	12	100	–	400	400	–
BPR-4	12	15	400	200	–	400	10
BPR-5	7	10	200	800	600	–	–
BPR-6	–	10	800	–	800	400	2
BPR-7	10	15	100	600	200	200	5
BPR-8	12	12	–	400	400	–	–
BPR-9	18	15	800	200	100	600	10
BPR-10	15	12	400	200	–	100	–
BPR-11	12	10	200	200	50	50	10
BPR-12	12	15	–	100	–	100	–
BPR-13	15	15	100	400	200	200	–
BPR-14	10	10	400	200	100	–	–
BPR-15	18	20	1000	800	800	800	20
BPR-16	12	18	600	–	50	100	–
BPR-17	15	15	400	100	100	400	5
BPR-18	–	10	200	50	–	–	10
BPR-19	15	–	800	100	200	200	10
BPR-20	10	12	200	200	100	200	–
BPR-21	7	10	100	200	100	100	2
BPR-22	5	10	200	200	200	100	-
BPR-23	7	5	200	50	100	50	2
BPR-24	10	10	100	100	50	200	-
BPR-25	15	7	50	200	50	100	-

**Table 2:** Screening of recovered endophytic microbes for plant growth regulating (PGR) attributes.

Endophytic Isolates	Production of Indole-3-Acetic Acid (µg mL^−1^)		ACC Deaminase Activity (μmol α-KB/mg Protein/h)		Siderophore Production				Production of	
	Qualitative	Quantitative	Qualitative	Quantitative	Qualitative	Halo Size (mm)	Salicylic Acid (µg mL^−1^)	2,3-DHBA (µg mL^−1^)	Ammonia	HCN
BPR-1	+	22.4 ± 1.7 (e)	–	–	+	16.2 ± 1.4 (c)	11.2 ± 1.6 (e)	21.2 ± 2.4 (c)	+	+
BPR-2	–	–	+	12.0 ± 1.8 (f)	++	20.3 ± 0.7(a)	12.8 ± 0.6 (e)	17.8 ± 0.8 (d)	+	–
BPR-3	++	40.2 ± 2.8 (c)	+	14.0 ± 1.3 (e)	+	10.3 ± 0.0 (e)	18.9 ± 0.72 (c)	21.4 ± 1.7 (c)	+	–
BPR-4	+	23.2 ± 3.2 (e)	+	9.2 ± 0.3 (g)	+	14.4 ± 2.2 (d)	13.2 ± 0.2 (e)	34.6 ± 2.6 (b)	++	+
BPR-5	+	17.8 ± 1.4 (f)	–	–	–	–	–	–	+	
BPR-6	+	24.2 ± 2.5 (d)	–	–	+	15.0 ± 2.1 (c)	17.3 ± 2.1 (c)	21.2 ± 0.81 (c)	++	
BPR-7	+	20.3 ± 2.0 (e)	+	21.1 ± 1.6 (c)	–	–	–	–	+	+
BPR-8	+	26.9 ± 3.0 (d)	+	18.4 ± 2.2 (d)	+	11.4 ± 1.4 (d)	9.6 ± 0.8 (g)	19.4 ± 2.1 (d)	++	+
BPR-9	+	25.2 ± 3.6 (e)	+	23.2 ± 2.2 (b)	++	19.6 ± 1.6 (a)	16.6 ± 3.1 (d)	38.9 ± 3.2 (b)	++	+
BPR-10	+	18..3 ± 1.3 (f)	+	17.1 ± 1.5 (d)	+	12.0 ± 0.3 (e)	23.4 ± 2.3 (b)	17.8 ± 1.7 (e)	++	+
BPR-11	+	17.6 ± 1.2 (f)	–	–	+	14.2 ± 1.8 (c)	31.3 ± 4.6 (a)	20.6 ± 0.9 (c)	+	+
BPR-12	–	–	+	12.1 ± 1.1 (f)	+	18.2 ± 2.3 (b)	23.4 ± 2.0 (b)	21.0 ± 4.6 (c)	+	+
BPR-13	++	36.5 ± 2.3 (c)	–	–	–	–	–	–	++	+
BPR-14	++	48.3 ± 3.1 (b)	+	14.0 ± 1.6 (e)	+	9.3 ± 0.8 (e)	25.2 ± 2.7 (b)	17.9 ± 2.3 (e)	++	–
BPR-15	+++	60.2 ± 3.7 (a)	++	27.2 ± 4.2 (a)	++	20.4 ± 2.6 (a)	33.9 ± 0.9 (a)	47.0 ± 2.1 (a)	+++	–
BPR-16	–	–	+	15.8 ± 2.0 (e)	+	14.0 ± 1.5 (c)	23.0 ± 2.4 (b)	23.8 ± 5.7 (c)	++	+
BPR-17	–	–	+	19.7 ± 3.1 (c)	–	–	19.7 ± 0.8 (c)	–	+	–
BPR-18	+	11.4 ± 0.38 (g)	–	–	+	16.0 ± 0.8 (b)	11.6 ± 2.3 (f)	18.6 ± 0.8 (e)	+	–
BPR-19	+	20.3 ± 2.0 (e)	+	20.4 ± 1.4 (c)	+	15.5 ± 2.3 (b)	10.7 ± 1.5 (f)	23.2 ± 3.5 (c)	+	+
BPR-20	+	21.4 ± 1.2 (e)	+	13.4 ± 1.7 (e)	+	11.7 ± 0.9 (e)	9.3 ± 0.5 (g)	17.8 ± 2.4 (e)	++	–
BPR-21	–	–	–	–	+	18.0 ± 2.0 (b)	12.4 ± 1.9 (f)	21.0 ± 4.6 (c)	+	–
BPR-22	–	–	–	–	+	18.0 ± 00 (b)	10.3 ± 0.9 (g)	17.9 ± 2.3 (e)	+	–
BPR-23	+	17.6 ± 1.2 (f)	–	–	+	15.2 ± 1.3 (c)	10.7 ± 1.5 (g)	21.4 ± 1.7 (c)	+	+
BPR-24	+	18..3 ± 1.3 (f)	–	–	–	–	–	–	++	–
BPR-25	+	16.0 ± 1.3 (f)	–	–	–	–	–	–	+	–

Here, ACC = 1-aminocyclopropane 1-carboxylate; α-KB = α-keto butyrate; NH_3_ = ammonia; HCN = hydrogen cyanide; 2,3-DHBA = dihydroxy benzoic acid; Single ‘–’, ‘+’ denotes no production and moderate production while ‘++’ and ‘+++’ represents the high and maximum production, respectively. Letters a, b, c, d etc. in parenthesis depicts that mean values are significantly different from each other according to Duncan’s multiple range test (DMRT).

**Table 3:** Screening of recovered endophytic isolates for nutrient solubilization efficiency.

Endophytic Isolates	P-Solubilization		K-Solubilization		Zn-Solubilization	
	Qualitative	Quantitative (Zone Diameter in mm)	Qualitative	Quantitative (Zone Diameter in mm)	Qualitative	Quantitative (Zone Diameter in mm)
BPR-1	++	18 ± 0.5 (b)	–	–	+	17.4 ± 2.3 (b)
BPR-2	–	–	–	–	–	–
BPR-3	–	–	+	14.5 ± 0.8 (c)	–	–
BPR-4	+	13 ± 1.5 (d)	–	–	+	12.1 ± 1.0 (e)
BPR-5	–	-	++	18.3 ± 1.3 (a)	–	-
BPR-6	+	10.4 ± 0.7 (e)	+	14.2 ± 0.7 (c)	+	14.02 ± 1.1 (d)
BPR-7	–	–	+	15.0 ± 0.6 (b)	–	–
BPR-8	+	12.0 ± 0.5 (d)	–	–	–	–
BPR-9	+	14.2 ± 1.2 (c)	–	–	–	–
BPR-10	++	18.5 ± 2.3 (b)	–	–	–	–
BPR-11	–	–	–	–	–	–
BPR-12	–	–	+	14.2 ± 0.4 (c)	+	14.0 ± 0.71 (d)
BPR-13	–	–	+	16.2 ± 0.56 (b)	+	15.1 ± 0.43 (d)
BPR-14	+	11.7 ± 0.6 (e)	+	12.5 ± 0.43 (d)	+	16.3 ± 0.4 (c)
BPR-15	++	20.0 ± 1.6 (a)	++	19.5 ± 0.8 (a)	++	20.1 ± 0.3 (a)
BPR-16	+	14.2 ± 0.7 (c)	–	–	–	–
BPR-17	+	11.3 ± 0.78 (e)	–	–	–	–
BPR-18	–	–	–	–	+	14.5 ± 2.1 (d)
BPR-19	–	–	–	–	+	16.0 ± 0.9 (c)
BPR-20	–	–	–	–	–	–
BPR-21	+	11.7 ± 0.6 (e)	+	15 ± 0.0 (b)	+	14.0 ± 0.0 (d)
BPR-22	+	14.2 ± 0.7 (c)	+	16.2 ± 0.56 (b)	+	16.0 ± 0.00 (c)
BPR-23	–	–	+	15.5 ± 0.6 (b)	–	–
BPR-24	+	12.0 ± 0.5 (d)	–	–	+	12.0 ± 0.71 (e)
BPR-25	–	–	–	–	+	14.0 ± 0.0 (d)

Values are means of three replicates. Mean values are significant at *p* ≤ 0.05. Means followed by the same letter are significantly different according to Duncan’s Multiple Range test. Single ‘–’, ‘+’ and ‘++’ denotes ‘no production’, ‘moderate production’ and ‘high production, respectively. Letters a, b, c, d etc. in parenthesis depicts that mean values are significantly different from each other according to Duncan’s multiple range test (DMRT).

## Data Availability

Not applicable.
